# Prevalence of Anaemia, Iron Deficiency, and Iron Deficiency Anaemia in Women of Reproductive Age and Children under 5 Years of Age in South Africa (1997–2021): A Systematic Review

**DOI:** 10.3390/ijerph182312799

**Published:** 2021-12-04

**Authors:** Eunice Turawa, Oluwatoyin Awotiwon, Muhammad Ali Dhansay, Annibale Cois, Demetre Labadarios, Debbie Bradshaw, Victoria Pillay-van Wyk

**Affiliations:** 1 Burden of Disease Research Unit, South African Medical Research Council, Cape Town 7505, South Africa; Oluwatoyin.Awotiwon@mrc.ac.za (O.A.); Ali.Dhansay@mrc.ac.za (M.A.D.); Annibale.Cois@mrc.ac.za (A.C.); Debbie.Bradshaw@mrc.ac.za (D.B.); victoria.pillay-vanwyk@mrc.ac.za (V.P.-v.W.); 2 Division of Human Nutrition, Faculty of Medicine and Health Sciences, Stellenbosch University, Tygerberg 7505, South Africa; 3 Department of Paediatrics and Child Health, Faculty of Medicine and Health Sciences, Stellenbosch University, Tygerberg 7505, South Africa; 4 Division of Health Systems and Public Health, Department of Global Health, Stellenbosch University, Cape Town 7505, South Africa; 5 Faculty of Medicine and Health Sciences, Stellenbosch University, Cape Town 7505, South Africa; dlabadarios@cybersmart.co.za; 6 Division of Epidemiology and Biostatistics, Department of Family Medicine and Public Health, University of Cape Town, Cape Town 7925, South Africa

**Keywords:** anaemia, iron deficiency, iron deficiency anaemia, prevalence, women of reproductive age, children under 5 years, systematic review

## Abstract

Using a systematic review method, the prevalence of anaemia, iron deficiency (ID), and iron deficiency anaemia (IDA) in women of reproductive age (WRA) and children under 5 years of age was obtained to inform priorities in health planning and policy in South Africa. We searched electronic databases for articles published between 1997 and 2021. A total of 713 articles were identified, of which 14 articles comprising 9649 WRA and 4085 children were included. Since most of the included studies were of low quality, we did not pool data in a meta-analysis due to heterogeneity (I^2^ > 75%). In WRA, anaemia prevalence ranged from 22.0% to 44.0%; ID from 7.7% and 19.0%; and IDA from 10.5% to 9.7%. The prevalence of anaemia in pregnancy was 29.0% to 42.7%; and 60.6% to 71.3% in HIV-infected pregnant women. Three national surveys reported anaemia in children at 28.9%, 10.7%, and 61.3%, respectively. Overall, among the children under 5 years old, anaemia was more prevalent in 1-year-olds (52.0%) compared to the other age groups. Between 2005 and 2012, ID increased by 3.8% and IDA decreased by 83.2% in children. Anaemia in WRA and children under 5 years in South Africa was a moderate public health concern. Therefore, interventions addressing anaemia should be intensified, and policies on iron supplementation and food fortification need to be revised and aligned to the WHO multiple micronutrient supplementation recommendations.

## 1. Introduction

Anaemia in women of reproductive age (WRA) and children under 5 years of age is a global public health problem associated with increased morbidity and mortality [1,2,3]. It is also an indicator of poor nutrition and wellbeing and is associated with poor cognitive and motor-neurological development in children [4]. Globally, about one-third (33%) of WRA (15–49 years) are anaemic, indirectly putting the nutrition and health of their children at risk [5]. The prevalence of anaemia in pregnant women varies widely across the regions of the world. In 2019, the global prevalence of anaemia was 36.5% among pregnant women, 29.6% in non-pregnant women, and 39.8% in children aged 6–59 months [4]. The highest prevalence of anaemia (56%) was found among pregnant women in low- and middle-income countries (LMICs), and the lowest (24.1%) was reported among pregnant women in South America [6,7]. Among World Health Organization (WHO) regions, Africa has the highest prevalence of anaemia in pregnancy (57%), followed by South-East Asia (48%) [3].

Generally, anaemia is classified according to its cause. Nutritional anaemia often results from inadequate intake of micronutrients, such as iron, folate, riboflavin, vitamins A, B12, and C required for blood formation. Other causes of anaemia include heavy menstruation, increased iron requirements during pregnancy and in growing children, chronic infections (such as tuberculosis, HIV, hookworm, and malaria), and poor iron absorption, transport, and storage, including haemoglobinopathies [8,9]. Nutritional iron deficiency (ID) is a disorder resulting from low dietary iron intake, increased iron demand and/or iron loss, as well as low bioavailability of iron from staple foods [10].

Worldwide, ID is a major contributor to the global burden of disease and affects children under the age of 5 years and WRA, including people living in the LMICs. ID accounts for about 50% of all cases of anaemia in women and remains a precursor to iron deficiency anaemia (IDA); which is one of the leading causes of years lived with disability [2]. In Africa, ID is common among WRA and children under 5 years of age due to the high level of poverty, and inadequate household food security. The Southern African Development Community (SADC) is one of the regions vulnerable to anaemia, with IDA in WRA ranging from 20% in Namibia to 54% in Mozambique, with adolescent girls and young women at higher risk of being affected [11].

Although the causes of anaemia are multifactorial, in Africa, IDA in WRA and children under 5 years of age is often due to the high level of poverty and inadequate household food security [12]; which is exacerbated by the high burden of HIV infection, poor nutrition knowledge, and inappropriate feeding practices.

A high prevalence of anaemia was reported among children under 5 years old in central and south Asia, and west Africa, where some countries reported an anaemia prevalence of over 70% in the general population [2]. The prevalence of anaemia was estimated at 59% in children under 5 years old in Tanzania, and the odds of being anaemic was higher in boys than in girls [13].

In women of childbearing age, anaemia is associated with the increased risk of adverse maternal (e.g., ante-partum and post-partum haemorrhage) and perinatal health outcomes (e.g., intrauterine foetal growth retardation, preterm birth, stillbirth), impaired physical health and cognitive development, and stunting in children [10,14]. HIV remains a major public health problem in South Africa, with over 53% of people living with HIV/AIDS being women and girls. This, together with other factors such as iron deficiency, pregnancy, or side effects of antiretroviral therapy (ART) and inherited blood disorders, increases the risk of anaemia in HIV-infected WRA [9].

The reduction of anaemia is a key priority of the World Health Assembly Global Nutrition Targets for 2025 [12], and of the Sustainable Development Goals. It is known that adequate dietary iron intake can significantly prevent and treat IDA, and the most cost-effective strategy to prevent and reduce anaemia includes food fortification with micronutrients, dietary supplementation, and oral iron supplementation. Therefore, the World Health Organization (WHO) recommended the use of these supplements as part of the standard prenatal care, and as a prophylactic measure to alleviate anaemia in pregnant women and children in high-burden regions [12]. In 2003, the South African government legislated the National Food Fortification Programme [15]. The legislation mandates the fortification of all cornmeal (maizemeal) and wheat flour with vitamin A, thiamin, riboflavin, niacin, folic acid, iron mineral, and zinc oxide to provide additional micronutrients and reduce the burden of anaemia and ID in the population [15]. Despite these investments, high anaemia prevalence has been reported consistently. Hoque et.al. (2007) [16] reported that 57.3% of pregnant women were anaemic, while another study reported an ID prevalence of 56.6% among the healthy female South African population [17].

The recent national South African Demographic and Health Survey (SADHS 2016) [18] estimated the prevalence of anaemia in WRA and children as 33.3% and 61.3%, respectively, whereas the national Food Consumption Survey-Fortification Baseline-1 (NFCS 2005) reported an anaemia prevalence of 29.4% and 28.9% for WRA and children under 5 years of age, respectively [19]. On the other hand, the South African National Health and Nutrition Examination Survey (SANHANES 2012) documented that 10.7% of children younger than 5 years in South Africa were anaemic [20]. South Africa is faced with the high burden of communicable diseases such as HIV/AIDS and tuberculosis, and non-communicable diseases which may aggravate anaemia in WRA and children under 5 years old [20].

Iron is essential for physiological and cellular processes. Reduced iron availability for red blood cell production could result in adverse health consequences. Therefore, a robust estimation of anaemia and the prevalence of ID is key to developing health and nutrition policies and strengthening available interventions for addressing anaemia and ID. To this end, this study systematically reviewed the available literature to determine the prevalence of anaemia, ID, and IDA among WRA (15–49 years), and children under 5 years of age in South Africa between 1997 and 2021. The findings from this review can help prioritize resource allocation for anaemia prevention and treatment, and inform policies aimed at achieving one of the Global Nutrition Targets 2025 of a 50% reduction in anaemia in WRA [12]. The review assessed the quality of available data on the prevalence of anaemia, ID, and IDA in WRA and children under 5 years of age, and estimated the prevalence of anaemia, ID, and IDA in WRA and children under 5 years of age in South Africa between 1997–2021.

## 2. Materials and Methods

The study protocol was registered with the International Prospective Register of Systematic Reviews (PROSPERO), (registration number: CRD42020193442). The Preferred Reporting Items for Systematic Reviews and Meta-Analyses (PRISMA) criteria for conducting systematic reviews and reporting were followed (Table S1) [21].

### 2.1. Search Strategy and Data Source

Using a comprehensive search strategy, we searched PubMed, Web of Science [ISI], Scopus, JSTOR, and CINAHL to identify articles reporting the prevalence of anaemia, iron deficiency, and iron deficiency anaemia in WRA and children younger than 5 years in South Africa, between 1997 and June 2021. The African Index Medicus (AIM) and online haematology and nutrition websites were searched for relevant articles. Search terms included keywords and medical subject headings [MeSH] such as “(anaemia OR anemia OR ferritin OR iron OR ferric OR serum ferritin) AND (women OR children OR infants OR under-5 OR under-5-years) AND (South Africa),” and synonym terms for each word. The search strategy used in PubMed is shown in Table S2. This was adapted for the other databases searched, and reference lists of retrieved articles were screened for additional studies. Linked articles in PubMed and experts in the field were contacted for additional eligible studies.

### 2.2. Operationalization of Variables

The diagnoses of anaemia, ID, and IDA in WRA were based on World Health Organization (WHO) guidelines, where anaemia was defined as a haemoglobin (Hb) concentration of <12.0 g/dL in nonpregnant women, and a haemoglobin concentration of <11.0 g/dL in pregnant women. The IDA diagnosis was established using the haemoglobin concentration of <12.0 g/dL plus a serum ferritin (SF) concentration of <15 µg/L in a physically healthy person with no apparent disease or infirmity. In children younger than 5 years, a haemoglobin concentration of <11.0 g/dL was indicative of anaemia, whereas a haemoglobin concentration of <11.0 g/dL and a serum ferritin concentration of <12 µg/L in the absence of infection or inflammation established a diagnosis of IDA [22,23], (Table S3).

### 2.3. Eligibility Criteria

Population-based surveys, cohort studies, and cross-sectional studies were included if they were conducted in South Africa and had more than 100 participants, regardless of the population group, socioeconomic, or educational background, and a prevalence estimate was reported according to the WHO diagnostic criteria [22,23]. All eligible articles that reported the prevalence of anaemia or ID or IDA in WRA (15–49 years) and children younger than 5 years between 1997–2021 were considered for inclusion. Randomised controlled trials, case reports, studies without original data, letters to the editor, and conference abstracts were excluded. Studies were excluded if they were not conducted among the South African population and did not contain numerical data that could be used to calculate prevalence. Studies that did not used the standardised WHO diagnostic criteria for assessment were also excluded.

### 2.4. Study Selection

Search outputs were exported into EndNote where duplicate articles were removed. Two reviewers (Eunice Turawa (ET) and Victoria Pillay-van Wyk (VPvW)) independently screened the titles and abstracts to identify potentially eligible articles. Full-text articles were retrieved and independently reviewed for inclusion. Discrepancies were resolved by discussion and/or consultation with the third reviewer (Oluwatoyin Awotiwon (OA)).

### 2.5. Quality Assessment and Data Extraction

The two reviewers (ET and VPvW) assessed each included article for risk of bias using a web-based standardized checklist for the systematic review of observational epidemiological studies, namely, the Burden of Disease Review Manager (BODRevMan) developed by the Burden of Disease Research Unit of the South African Medical Research Council [24]. The tool was adapted from the Risk of Bias Tool for population-based studies [25] and the Newcastle-Ottawa Scale for assessing the quality of non-randomized studies [26]. The BODRevMan consists of eight assessment domains that evaluate internal validity (appropriateness of case definition according to WHO diagnostic criteria [22,23], uncertainty of estimate, appropriateness of timing of outcome measurement, appropriateness of denominator and numerator used in calculating prevalence estimate, and confounders), and external validity (sampling frame and data collection, representativeness, and non-response bias). The overall quality score ranged from 1 to 20 (Table S4).

A study was rated as low risk of bias (quality score between 14 and 20), moderate (7 to 13), or high risk of bias (1 to 6) based on the total score. Other relevant information such as study author(s), year of publication, period in which the study was conducted and study setting, study design, and demographic characteristics of participants were also extracted. When information on any of the above was unclear, the authors of the article were contacted for additional information and clarity. Study response rates, prevalence estimates, and 95% confidence intervals (95% CIs) were extracted. When 95% CIs were not provided by the authors, we calculated their limits based on the reported sample size and prevalence using the normal approximation. Disagreements were resolved by discussion or consultation with the third reviewer.

### 2.6. Data Synthesis and Analyses

Prevalence estimates from individual studies were imported into STATA v14 statistical software (StataCorp, College Station, TX, USA) [27]. Given the variability in study design, setting, methodology, differences in socio demographic characteristics of participants, and the likely changes that may have occurred in prevalence estimates over the years (1997–2021), we anticipated a high degree of heterogeneity [28], which makes the data not amenable to meta-analysis. We, therefore, systematically presented the results from individual studies in a quantitative narrative discussion using figures and tables without pooling the individual estimates. For WRA, the results were reported in three subgroups: pregnant women, nonpregnant women, and HIV-infected pregnant women; results on children were reported under a separate table. Prevalence estimates and their 95% CIs were also reported for children under 5 years.

### 2.7. Patient and Public Involvement

This was a review of publicly available data: patients and public were not directly involved in the study.

## 3. Results

### 3.1. Study Results

**Figure 1:**
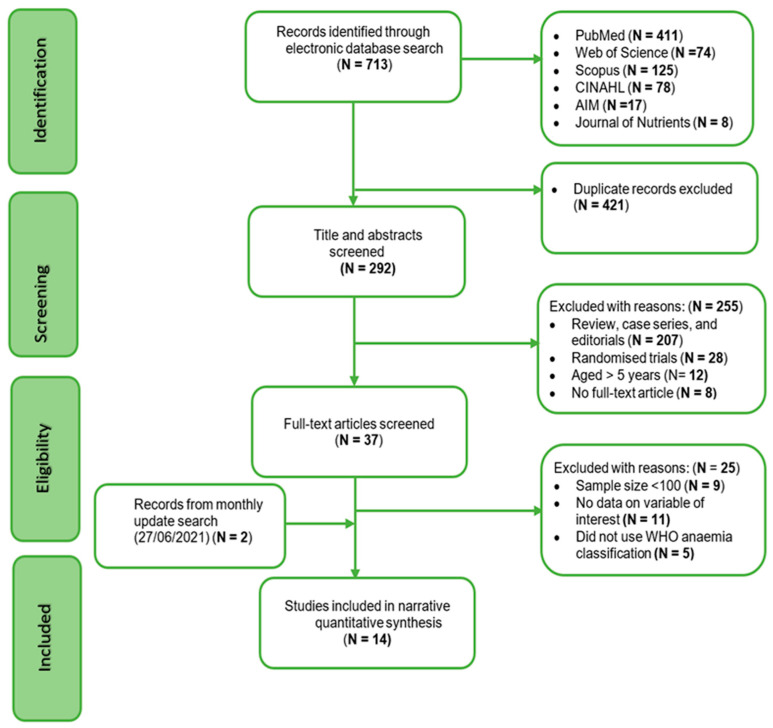
Flow diagram showing selection of studies for inclusion in the systematic review.

The article search and selection processes are outlined in Figure 1. In total, 713 articles were identified, and de-duplicated using EndNote engine. Titles and abstracts of 292 articles were screened for eligibility, and 37 full-text articles were retrieved, screened for inclusion, and 25 articles were excluded with reasons. Fourteen articles that met the inclusion criteria were included in this review. Figure 1 presents the flow diagram for the review process.

### 3.2. Characteristics of Included Studies Are Summarised in Tables 1 and 2

Of the 14 included studies, three (SADHS 2016, SANHANES 2012, and NFCS 2005) [18,19,20] were national surveys conducted across South African geographic locations and provinces. Of the remaining studies, seven were regional surveys [29,30,31,32,33,34,35], and four were cohort studies [36,37,38,39]. More than 50% were conducted among rural populations [29,30,31,32,33,36], while five studies were from urban settings [34,35,37,38,39].

Overall, ten studies reported prevalence of anaemia, ID, and IDA among WRA, while nine studies described prevalence in children under-5-years of age. Three studies measured prevalence of anaemia in pregnant women [18,35,39] and another three assessed anaemia in HIV-infected pregnant women [35,37,38]. Most of the studies were conducted among Black Africans [29,30,31,32,33,34,35,36,39]. Significant variability was observed between studies [29,30,31,32,33,34,35,36,37,38]. Methodologies were poorly reported in most studies, for example, consecutive sampling/convenience sampling was used in studies. Sample size calculation, response rate, and uncertainty level (e.g., standard deviation (SD) or standard error (SE)) were not reported in many of the local studies thereby limiting the rigorous assessment of whether the study methods were reliable, and the results could be trusted. The characteristics of the included studies are as presented in Table 1 and Table 2.

### 3.3. Prevalence of Anaemia, ID, and IDA in WRA (15–49 Years)

#### 3.3.1. Anaemia

**Figure 2:**
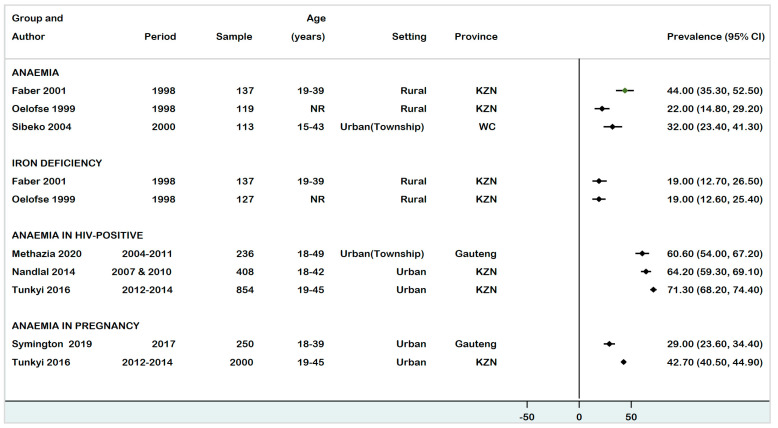
Forest plot of prevalence of anaemia and ID among WRA in South Africa (15–49 years), 1997–2021.

The prevalence of anaemia in WRA ranged from 22% (95% CI: 14.8–29.2%) to 44% (95% CI: 36.8–51.2%), with significant differences observed between studies with different designs, study periods (1997–2021), geographical locations, and characteristics of participants, which may have an important effect on the anaemia estimates (Figure 2). Amongst the local studies, the highest prevalence of 44% was reported in a study conducted in a rural community of KwaZulu-Natal [32].

The prevalence of anaemia in pregnant women ranged from 29% (95% CI: 23.6–34.4%) to 42.7% (95% CI: 40–45.4%) [18,35,39]. The highest prevalence of 42.7% was reported in Durban, KwaZulu-Natal province [35], while SADHS reported a lower prevalence of 39.1% [17].

Anaemia prevalence in HIV-infected pregnant women was investigated in three studies, which were all conducted in urban locations [35,37,38]. A study conducted in Johannesburg estimated anaemia in HIV-infected pregnant women as 60.6% [37]; whereas Nandlal et al. (2014) and Tunkyi et al. (2016) reported a much higher prevalence of 64.2% [38] and 71.3% [35], respectively, in KZN province.

#### 3.3.2. Iron Deficiency

Oelofse et al. (1999) and Faber et al. (1998) reported a similar prevalence of 19% (95% CI: 12.5–25.5%) [30,33], among Black African WRA residing in rural KZN, while two national surveys recorded a much lower ID prevalence of 7.7% (95% CI: 6.6–8.8%) in 2005 [19], and 5.9% (95% CI: 13.0–17.0%) in 2012 among WRA [20] signifying a 23.4% decrease in ID prevalence (Figure 2).

#### 3.3.3. Iron Deficiency Anaemia

None of the local studies reported on the burden of IDA in WRA.

#### 3.3.4. National Prevalence Estimates of Anaemia, ID, and IDA in WRA (1997−2021)

**Figure 3:**
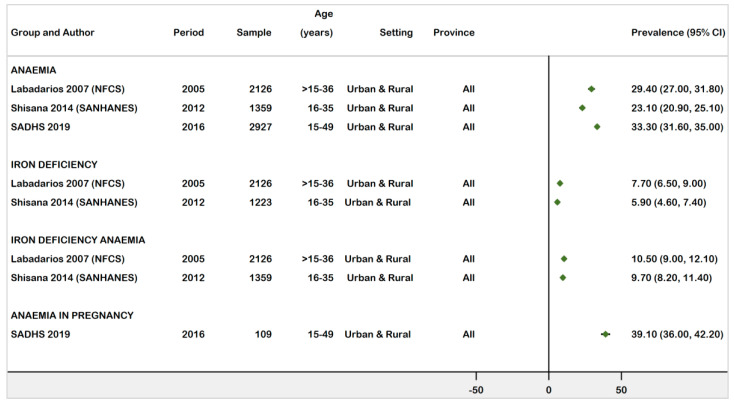
Forest plot of national prevalence of anaemia, ID, and IDA in WRA in South Africa (15–49 years), 1997–2021.

Figure 3 displays the national prevalence estimates for anaemia, ID, and IDA. The prevalence of anaemia in non-pregnant women ranged from 23.1% to 33.3%, and 39.1% in pregnant women [18 −20]. Iron deficiency was reported by two surveys conducted in 2005 and 2012. Prevalence estimates of 7.7% (95% CI: 6.5%–9.0%) and 5.9% (95% CI: 4.6%–7.4%), respectively, were recorded for WRA, whereas the prevalence burden of IDA was estimated at 10.5% (95% CI: 9.0–12.1%) and 9.7% (95% CI: 8.2–11.4%).

### 3.4. Prevalence Estimate in Children under 5 years

#### 3.4.1. Prevalence of Anaemia, ID, and IDA in Infants Age 1 Month -13 Months

**Figure 4:**
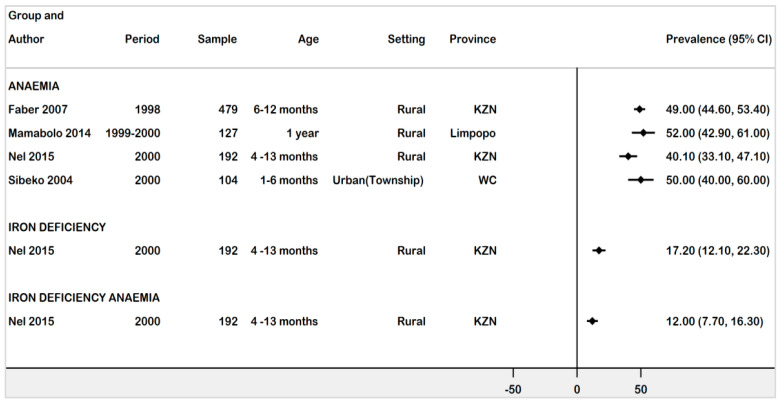
Forest plot of prevalence of anaemia, ID, and IDA in infants aged 1–13 months in South Africa, 1997–2021.

Four studies conducted between 1998 and 2000 investigated the prevalence of anaemia, ID, and IDA in infants age 1 month–13 months [29,32,34,36]. The prevalence of anaemia ranged from 40.1% (95% CI: 33.1–47.1%) to 52% (95% CI: 42.9–61.0%). The highest prevalence of 52% (95% CI: 42.9–61.0%) was reported by a study conducted in a rural community of Limpopo. A study conducted in an urban township of Western Cape (Sibeko 2004) estimated the prevalence of anaemia in children age 1–6 months as 50% (95% CI: 40–60%). Except for Sibeko 2004, all studies were conducted in various rural communities. A study conducted in KZN assessed ID and IDA in children age 4 -13 months as 17.2% (95% CI: 12.1–22.3%) and 12.0% (95% CI: 7.7–16.3%), respectively (Figure 4).

#### 3.4.2. Prevalence of Anaemia, ID, and IDA in Children Age 24 -59 Months

**Figure 5:**
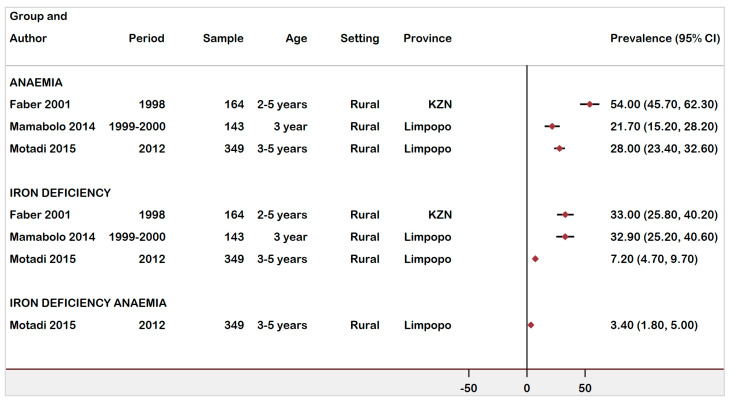
Forest plot of prevalence of anaemia, ID, and IDA in children age 24 months–59 months in South Africa, 1997–2021.

The prevalence of anaemia in Black African children was reported in three studies [30,31,36], all conducted in Limpopo and KZN province. Anaemia prevalence ranged from 21.7% (95% CI: 15.2–28.2%) to 54% (95% CI: 46.7−62.3%). These studies also reported on ID prevalence, with the highest prevalence found in children from KZN (33%) and in Limpopo (32.9%). Iron deficiency anaemia was estimated as 3.4% (95% CI: 1.8–5.0%) in children ages 36–59 months old (Figure 5).

#### 3.4.3. National Prevalence of Anaemia, ID, and IDA Children Ages 0 -5 Years Old

**Figure 6:**
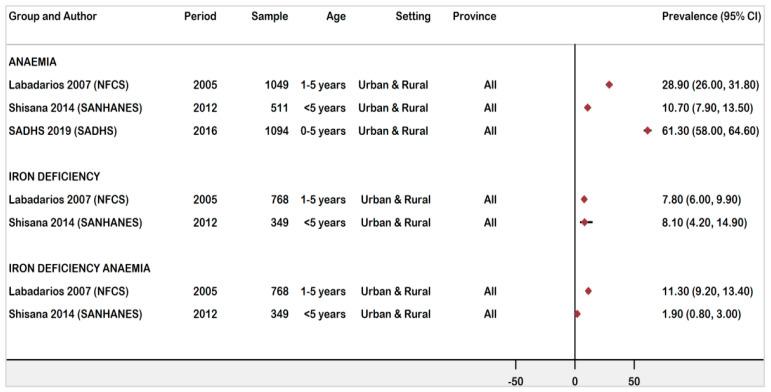
Forest plot of national prevalence of anaemia, ID, and IDA in children ages 0–5 years old in South Africa, 1997–2021.

Three national surveys [18,19,20] reported anaemia prevalence as 61.3%, 10.7%, and 28.9%, respectively. The prevalence of anaemia declined markedly from 28.9% (95% CI: 26.0–31.8%) in 2005 to 10.7% (95% CI: 7.9–13.5%) in 2012. However, the recent SADHS 2016 reported a marked increase in anaemia prevalence in children ages 0 -5 years old 61.3% (95% CI: 58.0–64.6%) [18] (Figure 6).

Two national surveys [19,20] showed a marginal increase in ID between 2005 (7.8%) and 2012 (8.1%), and a steady and significant decrease in IDA from 11.3% in 2005 to 1.9% in 2012.

### 3.5. Anaemia Prevalence in Rural vs. Urban Settings in Children under 5 Years

The NFCS 2005 survey reported a higher anaemia prevalence (32.4%) in the urban population compared to the rural population (23.9%) [19]; whereas SANHANES 2012 [20], showed a negligible difference in the prevalence of anaemia in rural vs. urban areas. Between 2005 and 2012, anaemia prevalence reduced by 41% and 59.9% in rural and urban populations, respectively [20].

The recent SADHS 2016 survey reported a sharp increase in anaemia prevalence to 60.4% and 62.2% [18] for rural and urban under 5-year-olds, respectively (Figure S5).

### 3.6. Comparison of Prevalence of Anaemia in WRA and Children under 5 Years of Age

**Figure 7:**
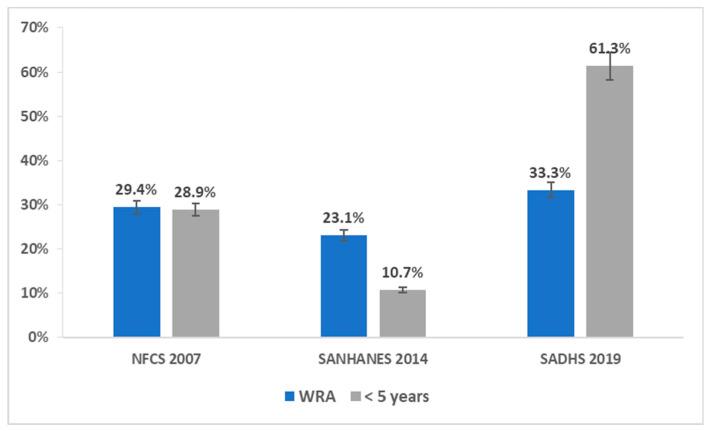
Comparison of anaemia prevalence in WRA and children under 5 years of age in South Africa, 1997–2021.

SANHANES 2012 showed a 6.3 and 18.2 percentage points decrease in anaemia prevalence in WRA and under 5-year-olds, respectively. In contrast, the recent SADHS 2016 showed a sharp increase in anaemia prevalence for both groups, with a higher estimate for children (61.3%) than for WRA (33.3%) (Figure 7).

## 4. Discussion

Anaemia and ID are a major public health problem in WRA and children under 5 years old. This study assessed the prevalence of anaemia, ID, and IDA in WRA and the under 5-year-olds in the South African population between 1997 and 2021. To our knowledge, this is the first systematic review of this important topic in South Africa. A standardised risk of bias assessment tool was used to evaluate the quality of the included studies. We were unable to perform the planned meta-analysis and subgroup analysis due to substantial heterogeneity in the included studies. About two-thirds of the studies were conducted over two decades ago and were of low methodological quality. The participants were mostly Black Africans from rural and underserved communities, limiting the generalisability of the study findings.

The prevalence of anaemia in non-pregnant WRA ranged from 22% to 44%, 29% to 42.7% in pregnant women, 60.6% to 71.3% in HIV-infected pregnant women, and 10.7% to 61.3% in children younger than 5 years. It is noteworthy that the wide variabilities in year of study, and the changes that may have occurred in prevalence estimates over the years, including differences in study design, geographical location (mountainous areas), small unrepresentative samples, and low methodological quality may have biased (over/underestimated) the true prevalence in the included studies.

According to the WHO classification, the current anaemia estimates in WRA and the under 5-year-olds in South Africa are of moderate public health importance [40], despite the National Food Fortification Programme and iron-folate supplementation. This is indicative of the urgent need for more effective interventions to reduce the burden of anaemia in these populations.

Undoubtedly, the devastating impact of HIV and TB infections against a backdrop of inadequate nutrition (vitamin B12 or folic acid), and poverty could have exacerbated the prevalence of anaemia in WRA [20], particularly in HIV-infected WRA where IDA can be associated with disease progression and survival rate [41].

### 4.1. Prevalence of Anaemia, ID, and IDA in WRA

The current findings on anaemia in WRA are consistent with the WHO reported estimate (30.5%) in South Africa [4,42], and the prevalence reported in Central Asia, the Middle East, and North Africa [2,43]. However, the prevalence of anaemia in WRA in South Africa is lower than that reported in South Asian and South-East Asian countries, where the prevalence of anaemia in WRA was 52.5% [44]. The prevalence of anaemia in pregnancy is consistent with that of the 2010–2013 South African National Maternal Mortality Report [45]. Many WRA from LMICs are unable to meet their iron requirement during pregnancy due to pre-pregnancy inadequate dietary iron consumption and low socio-economic status. In the context of HIV infection, anaemia and ID are independent markers of HIV disease progression [46,47].

There was a significant reduction of 6.3 percentage points in anaemia prevalence among WRA between 2005 and 2012, and a decrease of 23.4% in ID prevalence, which may partly be due to the Food Fortification Programme and the routine Iron Folate Supplementation Programme. However, the prevalence of IDA appears static over the study period, which may be related to the suboptimal consumption of dietary iron and micronutrients required for haemoglobin formation.

### 4.2. Prevalence of Anaemia, ID, and IDA in Children under 5 Years

The trend of anaemia in children under 5 years in South Africa is consistent with estimates reported for South Asia and Oceania [2,42]. The current report is also consistent with the anaemia prevalence (59%) reported in children under 5 years old in Tanzania, with local variation ranging from 53% to 71% in anaemia burden [13].

Between 2005 and 2012, the prevalence of anaemia in children under 5 years of age decreased by 18.2 percentage points. The recent SADHS 2016 shows a much higher prevalence of anaemia (61.3%); however, the authors advised caution on the estimate. Blood samples were taken from a finger prick or heel prick, whereas all other national surveys used venous blood. Literature suggests that blood sample type (capillary samples vs. venous samples), humidity, HemoCue^®^ model, the environmental conditions (high temperature), analytical models, and adjustment/non-adjustment for altitude, may overestimate/underestimate the haemoglobin measurement and observed anaemia prevalence [48].

The Malawi Demographic and Health Survey (DHS 2015/2016) and Micronutrient Status Survey (MNS) BRINDA, 2015/16, utilized capillary and venous blood samples to investigate anaemia in children. In both surveys, haemoglobin concentrations were assessed with a portable hemoglobinometer (HemoCue 301). The results showed a significant difference in reported anaemia prevalence (61% vs. 30%) [49]. In view of the above, we suspect that the SADHS 2016 anaemia prevalence results may have been influenced by the blood sample type.

Overall, the prevalence of anaemia is highest in children ages 1 month to 30 months old compared to all other age groups in the South African population. This correlates to a recent finding in Namibia, where a higher anaemia prevalence was observed among children ages 6–24 months, and that anaemia prevalence decreases with age, possibly because older children can consume more food rich in iron compared to infants [50].

Another important observation was the inclusion of children below the age of 6 months as participants [32,34]. One of the studies had strict inclusion criteria resulting in highly selective healthy participants [32], which could have blurred the true prevalence of anaemia in the studied group. Other studies from African countries showed that younger children of less than 2 years, boys, and children from poorer households have an increased risk of anemia [13,50]. Poor access to health services and inadequate feeding remains important causes of anaemia which could result in impaired physical and intellectual capacity in children later in life.

Between 1998 and 2012, there was a 26-percentage point reduction in ID in children between the ages of 24 −42 months old. The decrease in ID may partly be due to the National Food Fortification Intervention and the Micronutrient Supplementation programmes in the country. Similarly, a 9.4 percentage point decrease was observed in IDA between 2005 and 2012, although it is uncertain whether the observed decline represents a true trend because the IDA response rate in SANHANES 2012 was very low (20%).

In view of the adverse effect of anaemia and ID on WRA and child development, national policies on nutrition and factors exacerbating anaemia and ID risk should be prioritised [50]. Although food fortification and supplementation are not a panacea for all types of anaemia, evidence abounds that food fortification on a large scale can reduce anaemia in populations when it is effectively implemented. A systematic review and meta-analysis from LMICs found that anaemia decreased by about 34% after large-scale food fortification [51]. In addition to low compliance with food fortification standards by the food industry [52], low household food security, poverty, and disruption in economic conditions can also aggravate anaemia prevalence [53]. In the meantime, improved socio-economic conditions, strengthening healthcare quality, and infection prevention and control, and improved access to nutritional counselling, could lessen the burden of anaemia in the country [54].

### 4.3. Strengths and Limitations of the Review

The strength of the review is that it is the first systematic review assessing the prevalence of anaemia, ID, and IDA in WRA and children under 5 years of age in the South African population, between 1997 and 2021. In total, 9649 WRA and 4085 children were included in the review. Most of the blood samples were venous samples, except for SADHS 2016, which used capillary samples. The PRISMA guidelines, and a standardized risk of bias assessment tool were used to evaluate the quality of included studies, and data were systematically extracted and presented in tables and graphs.

Limitations of this review include substantial heterogeneity (I^2^ > 75%) in the included studies, which prevented the planned meta-analysis and subgroup analysis. In addition, most of the included studies (two-thirds) were conducted more than two decades ago and were of low methodological quality as the sample size calculation, response rate, and uncertainty levels (i.e., standard deviation (SD) or standard error (SE) or 95% confidence interval (95% CI)) were not reported in most of the local studies. The participants were mostly Black Africans from rural areas for many of the studies, limiting the generalisability of the study findings.

## 5. Conclusions

Currently, it is unlikely that South Africa will achieve the global nutritional target of a 50% reduction in anaemia in WRA by 2025. The magnitude of anaemia in WRA and children <5 years of age requires urgent combined interventions and cost-effective measures to address the underlying causes of anaemia.

Advancing towards the World Health Assembly Global Nutrition Targets for 2025 of a 50% reduction in anaemia in WRA will require addressing both the direct and indirect determinants of anaemia, e.g., poverty and the infectious disease burden in WRA and children under 5 years old. It is also crucial that policymakers, economists, and programme managers involved in the designing and implementation of food and nutrition programmes decide on appropriate nutrition actions required to prevent and control anaemia and ID in WRA and children under 5 years old in South Africa. In addition to strong political commitment, strengthening partnerships with agricultural sector and the health system can can promote and enhance nutrition in WRA and children under 5 years old. Similarly, empowerment of young women and adolescent girls through education on improved and adequate nutrition and adherence to supplementation are necessary for achieving the Global Nutrition Target of a 50% anaemia reduction in WRA. The existing prenatal supplementation strategies, food fortification with iron, folic acid and essential micronutrients should be reviewed to identify gaps in the Supplementation Programme and delivery channels. Furthermore, strategies to improve iron bioavailability and absorption, as well as food security at the household level are needed, alongside the effective monitoring of food industries to ensure food fortification is according to standard. We also recommend that the country’s policies and programmes on nutrition need to be reviewed with particular attention paid to WRA, children under 5 years old, and WRA living with HIV/AIDS, while multiple micronutrient supplementation (MMS) is intensified [55,56].

## Figures and Tables

**Table 1:** Characteristics of included studies on anaemia, ID, and IDA in WRA (15–49 years) in South Africa, 1997–2021.

Study ID	Study Design	Study Period	Setting	Population	Age (Year)	Condition	Case Definition	Sample (N)	Sample (n)	Prevalence		Risk of Bias Score
										%	95% CI	
Faber (2001) [30]	Cross-sectional	1998	Rural	Women	19–39	Anaemia	Hb < 12 g/dL	137	60	44	35.3–52.5	Low
						ID	SF < 15μg/L		26	19	12.7–26.5	
Labadarios (2007) [19] (NFCS)	Population-based survey	2005	National	Women	16–36	Anaemia	Hb < 12 g/dL	2126	625	29.4	27.0–31.8	Low
						IDA	Hb < 12 g/dL & SF < 15μg/L		223	10.5	9.0–12.1	
						ID	Hb ≥ 12 g/dL & SF < 15μg/L		164	7.7	6.5–9.0	
Methazia (2020) [37]	Cohort	2004–2011	Urban	HIV-infected pregnant	18–49	Anaemia	Hb < 10.0–10.9 g/dL	236	142	60.6	54.0–67.2	Low
Nandlal (2014) [38]	Cohort	2007 & 2010	Urban	HIV-infected pregnant	18–42	Anaemia	Hb < 11 g/dL	408	262	64.2	59.3–69.1	Moderate
Oelofse (1999) [33]	Cross-sectional	1998	Rural	Black African	NR	Anaemia	Hb < 12 g/dL	119	26	22	14.8–29.2	Moderate
						ID	Hb > 12 g/dL & SF < 15μg/L	127	24	19	12.6–25.4	
Shisana (2014) [20] (SANHANES)	Population-based survey	2012	National	Women	16–35	Anaemia	Hb < 12 g/dL	1359	313	23.1	20.9–25.1	Low
						ID	Hb > 12 g/dL & SF < 15μg/L	1223	72	5.9	4.6–7.4	
						IDA	Hb ≤ 12 g/dL & SF ≤ 15μg/L	1359	132	9.7	8.2–11.4	
Sibeko (2004) [34]	Cross-sectional	2000	Urban	Black African	15–43	Anaemia	Hb < 12 g/dL	113	36	32	23.4–41.3	Low
South Africa Demographic & Health Survey (2019) [18] (SADHS)	Population- based survey	2016	National	women	15–49	Anaemia	Hb < 12 g/dL	2927	975	33.3	31.6–35.0	Low
				Pregnant women			Hb < 11 g/dL	109	42	39.1	36–42.2	
Symington (2019) [39]	Cohort	2017	Urban	Pregnant women	18–39	Anaemia	Hb < 11 g/dL	250	73	29	23.6–34.4	Moderate
Tunkyi (2016) [35]	Cross-sectional	2012–2014	Urban	Pregnant women	19–45	Anaemia	Hb < 11 g/dL	2000	854	42.7	40.5–44.9	Moderate
				HIV-infected pregnant				854	609	71.3	68.2–74.4	

**Table 2:** Characteristics of included studies on anaemia, ID, and IDA for children under 5 years of age in South Africa, 1997–2021.

Study ID	Study Design	Study Period	Setting	Population	Condition	Age	Case Definition	Sample	Sample	Prevalence		Risk of Bias Score
								(N)	(n)	%	95% CI	
Faber (2001) [30]	Cross-sectional	1998	Rural		Anaemia	2–5 years	Hb < 11 g/dL		88	54	45.7–61.5	Low
				African children	ID		SF < 12μg/L	164	54	33	25.8–40.7	
Faber (2007) [29]	Cross-sectional	1998	Rural	Children	Anaemia	6–12 months	Hb < 11 g/dL	498	246	49	44.6–53.4	Moderate
Labadarios (2007) [19] (NFCS-FB-1)	Population-based survey	2005	National		Anaemia		Hb < 11 g/dL	1049	303	28.9	26.0–31.8	Low
				Children	ID	1–5 years	Hb ≥ 11 g/dL & SF 12μg/L	821	151	7.8	6.0–9.9	
					IDA		Hb < 11 g/dL& SF < 12μg/L	768	87	11.3	9.2–13.4	
Mamabolo (2014) [36]	Cohort	1999–2000	Rural		Anaemia	1 year	Hb < 11 g/dL & SF < 12μg/L	127	66	52	42.9–60.9	Moderate
				Children		3 years		143	31	21.7	15.2–28.2	
					ID	3 years	SF < 12μg/L	143	47	32.9	25.2–40.6	
Motadi (2015) [31]	Cross-sectional	2012	Rural	Children	Anaemia	3–5 years	Hb < 11 g/dL	349	98	28	23.4–32.6	Moderate
					ID		SF < 12μg/L or TSAT < 15%		25	7.2	4.7–9.7	
					IDA		Low Hb & TSAT or SF or both		12	3.4	1.8–5.0	
Nel (2015) [32]	Cross-sectional				Anaemia	4–13 months	Hb < 11 g/dL		77	40.1	33.1–47.1	Low
		2000	Rural	African children	ID		SF < 12μg/L	192	33	17.2	12.1–22.3	
					IDA		Hb < 11 g/dL & SF < 12μg/L		23	12	7.7–16.3	
Shisana (2014) [20]	Population-based survey	2012	National	Children	Anaemia	< 5 years	Hb < 11 g/dL	511	56	10.7	7.9–13.5	Low
(SANHANES)					ID		Hb ≥ 11 g/dL & SF < 12μg/mL	349	28	8.1	5.4–11.4	
					IDA		Hb < 11 g/dl & SF < 12μg/L		7	1.9	0.8–4.0	
Sibeko (2004) [34]	Cross-sectional	2000	Urban	African children	Anaemia	1–6 months	Hb < 11 g/dL	104	52	50	40.0–60	Low
South Africa Demographic & Health Survey (2019) [18] (SADHS)	Population-based survey	2016	National	Children	Anaemia	0–5 years	Hb < 11 g/dL	1094	667	61.3	58.0–64.6	Low

## Data Availability

No additional data available.

## Supplementary Materials

The following are available online at https://www.mdpi.com/article/10.3390/ijerph182312799/s1, Table S1: PRISMA 2009 Checklist, Table S2: PubMed search strategy, Table S3: Anaemia and Iron deficiency anaemia diagnostic guidelines, Table S4: Quality assessment criteria for prevalence studies (BODRevMan), Figure S1: Rural vs. Urban anaemia prevalence in children under 5 years of age in South Africa.
