# Supplementary material for: Prevalence of Anaemia, Iron Deficiency, and Iron Deficiency Anaemia in Women of Reproductive Age and Children under 5 Years of Age in South Africa (1997–2021): A Systematic Review
Source: Int J Environ Res Public Health. 2021 Dec 4;18(23):12799. doi: 10.3390/ijerph182312799 (PMC8656986; doi:10.3390/ijerph182312799)
Supplement: Supplementary file 1 [file ijerph-18-12799-s001.zip › ijerph-1463162-supplementary.pdf]

Table S1. PRISMA 2009 Checklist.

Section/topic	#	Checklist item	Reported on page #
**TITLE**			
Title	1	Identify the report as a systematic review, meta-analysis, or both.	1
**ABSTRACT**			
Structured summary	2	Provide a structured summary including, as applicable: background; objectives; data sources; study eligibility criteria, participants, and interventions; study appraisal and synthesis methods; results; limitations; conclusions and implications of key findings; systematic review registration number.	1-2
**INTRODUCTION**			
Rationale	3	Describe the rationale for the review in the context of what is already known.	2-3
Objectives	4	Provide an explicit statement of questions being addressed with reference to participants, interventions, comparisons, outcomes, and study design (PICOS).	3
**METHODS**			
Protocol and registration	5	Indicate if a review protocol exists, if and where it can be accessed (e.g., Web address), and, if available, provide registration information including registration number.	3
Eligibility criteria	6	Specify study characteristics (e.g., PICOS, length of follow-up) and report characteristics (e.g., years considered, language, publication status) used as criteria for eligibility, giving rationale.	3-4
Information sources	7	Describe all information sources (e.g., databases with dates of coverage, contact with study authors to identify additional studies) in the search and date last searched.	3
Search	8	Present full electronic search strategy for at least one database, including any limits used, such that it could be repeated.	3 and Table S1
Study selection	9	State the process for selecting studies (i.e., screening, eligibility, included in systematic review, and, if applicable, included in the meta-analysis).	4, Table S3
Data collection process	10	Describe method of data extraction from reports (e.g., piloted forms, independently, in duplicate) and any processes for obtaining and confirming data from investigators.	4, Table S4
Data items	11	List and define all variables for which data were sought (e.g., PICOS, funding sources) and any assumptions and simplifications made.	8 and 9; Figure 3 and 4

Risk of bias in individual studies	12	Describe methods used for assessing risk of bias of individual studies (including specification of whether this was done at the study or outcome level), and how this information is to be used in any data synthesis.	4-5
Summary measures	13	State the principal summary measures (e.g., risk ratio, difference in means).	7-10
Synthesis of results	14	Describe the methods of handling data and combining results of studies, if done, including measures of consistency (e.g., I^2) for each meta-analysis.	3, 8 and 9
**Section/topic**	**#**	**Checklist item**	**Reported on page #**
Risk of bias across studies	15	Specify any assessment of risk of bias that may affect the cumulative evidence (e.g., publication bias, selective reporting within studies).	5 and 6
Additional analyses	16	Describe methods of additional analyses (e.g., sensitivity or subgroup analyses, meta-regression), if done, indicating which were pre-specified.	10
**RESULTS**			
Study selection	17	Give numbers of studies screened, assessed for eligibility, and included in the review, with reasons for exclusions at each stage, ideally with a flow diagram.	6, figure 1
Study characteristics	18	For each study, present characteristics for which data were extracted (e.g., study size, PICOS, follow-up period) and provide the citations.	6, Table S5 and S6
Risk of bias within studies	19	Present data on risk of bias of each study and, if available, any outcome level assessment (see item 12).	Table S5 and Table S6
Results of individual studies	20	For all outcomes considered (benefits or harms), present, for each study: (a) simple summary data for each intervention group (b) effect estimates and confidence intervals, ideally with a forest plot.	8 and 9
Synthesis of results	21	Present results of each meta-analysis done, including confidence intervals and measures of consistency.	7-10
Risk of bias across studies	22	Present results of any assessment of risk of bias across studies (see Item 15).	Table S5 and Table S6
Additional analysis	23	Give results of additional analyses, if done (e.g., sensitivity or subgroup analyses, meta-regression [see Item 16]).	9 and 10
**DISCUSSION**			
Summary of evidence	24	Summarize the main findings including the strength of evidence for each main outcome; consider their relevance to key groups (e.g., healthcare providers, users, and policy makers).	11-12
Limitations	25	Discuss limitations at study and outcome level (e.g., risk of bias), and at review-level (e.g., incomplete retrieval of identified research, reporting bias).	12
Conclusions	26	Provide a general interpretation of the results in the context of other evidence, and implications for future	12-13

		research.	
**FUNDING**			
Funding	27	Describe sources of funding for the systematic review and other support (e.g., supply of data); role of funders for the systematic review.	13

Table S2. *PubMed search strategy* ***The PubMed search strategy was adapted to search other databases.**

**Search Number**	**Keywords Combination**
**#7**	Search (#6 NOT (animals[mh]NOT humans[mh]) AND ("1997/01/01"[Date-Publication]: "2021/06/27"[Date - Publication])
**#6**	Search (#4 AND #5)
**#5**	Search (South Africa[mh]OR South Africa*[tiab] OR RSA [tiab] OR Africa, Southern[mh:noexp] OR Southern Africa[tiab])
**#4**	#1 AND #2 AND #3
**#3**	Search (women(mh] OR female[mh] OR children[mh] OR infants[mh] OR toddler OR babies[mh])
**#2**	Search (Anemia [mh] OR Anemia* OR anaemia* OR anemic OR anaemic)
**#1**	Search (Iron [mh] OR ferrous OR ferric OR ferritin OR iron)

Table S3. Anaemia and Iron deficiency anaemia diagnostic guidelines (WHO 2011 & 2020).

[1]Adapted from: World Health Organization. Haemoglobin concentrations for the diagnosis of anaemia and assessment of severity. World Health Organization, 2011. WHO/NMH/NHD/MNM/11.1. https://www.who.int/vmnis/indicators/haemoglobin/en/.

Population group	Hb g/dL (apparently healthy persons)	SF µg/L (apparently healthy persons)[3]	SF µg/L (Individuals with infection/inflammation)
**Infants (0–23 months)**	-	<12[2]	<30[2]
**Children <5 years of age**	<11.0[1]	<12[2]	<30[2]
**Children (5 to 11 years)**	11.5[1]	<15[2]	<70[2]
**Adolescents (10 to <20years)**	-	<15[2]	<70[2]
**Adults (20–59 years)**		<15[2]	<70[2]
**Older persons (60+ years)**	-	<15[2]	<70[2]
**Pregnant females**	<11.0[1]	<15 (first trimester)[2,*]	-
**Non-pregnant females**	<12.0[1]	<15[1]	-

[2]World Health Organization (2020). WHO guideline on use of ferritin concentrations to assess iron status in individuals and populations.

*Several physiological changes occurring in pregnancy that may contribute to the variation in thresholds of iron deficiency in pregnancy as defined by serum ferritin. Physiological rise in acute phase proteins secondary to pregnancy; second trimester plasma volume expansion; and changes in inflammatory measures in the final trimester of pregnancy.

Table S4. Quality assessment criteria for prevalence studies.

DOMAIN	CRITERIA	tion of DESCRIPTION OF ASSESSMENT	SCORE
**External validity**	Representativeness	Was a sample size calculation conducted and is it adequate?	**1**
		Is the target population a close representation of the national population in relation to relevant variables?	**1**
		Was the sampling frame a true or close representation of the population?	**1**
		Was a form of random selection used to select the sample? Was the sampling method appropriate for the research question?	**2**
	Non response bias	Were there similarities between participants and non-participants in relation to demographic characteristics?	**1**
		Was the overall/response rate of the study reported?	**1**
		What was the overall/response rate for the study?	**1**
		Was the overall/response rate adequate for the study? Excellent ≥80%, Average 60-79%, Poor <60%	**1**
**Internal validity**	Case definition	Were the cases classified using the ICD codes or was an acceptable case definition used? What is the case definition?	**1**
	Data Collection	Were the study instruments used to measure the parameter of interest shown to have reliability and validity in this study or a previous study?	**2**
		Were data collected directly from the participants or if a proxy (a representative of the participant) was used, was it appropriate?	**1**
		Was the same method used for data collection for all participants for the condition of interest? If a different method was used, was it adequate?	**1**
	Uncertainty of estimation	Was the parameter of interest reported with uncertainty, i.e. Standard Deviation (SD) or	**1**

		Standard Error (SE) or 95% Confidence Interval (CI)?	
	Appropriateness of time factor for outcome measure	Was the length of recall period for the parameter of interest appropriate to ascertain outcome/exposure?	**2**
	Appropriateness of numerator and denominator in calculation of estimate	Were the numerator and the denominator for the parameter of interest appropriate? If not, can these be extracted to recalculate the parameter of interest?	**2**
	Confounding	Were potential confounding factors sought and controlled for in the study?	**1**
**Total score**			**20**

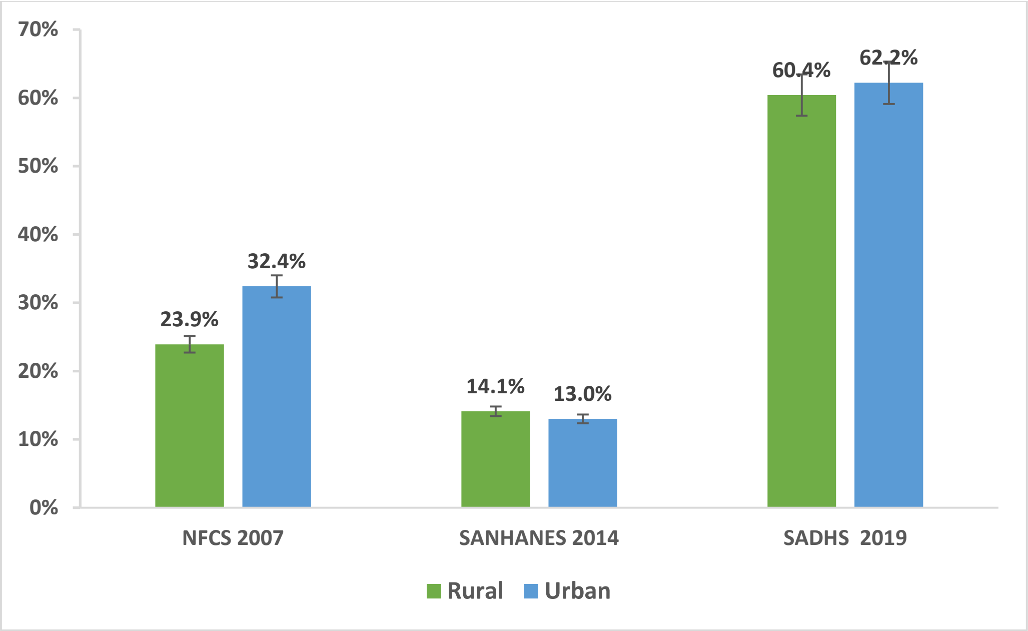

Figure S1. Rural vs Urban anaemia prevalence in children under 5 years of age in South Africa, 1997–2021.
